# Examination of resident characteristics associated with interest in primary care and identification of barriers to cross-cultural care

**DOI:** 10.1186/s12909-021-02669-w

**Published:** 2021-04-19

**Authors:** Sara Abrahams, Eun Ji Kim, Lyndonna Marrast, Omolara Uwemedimo, Joseph Conigliaro, Johanna Martinez

**Affiliations:** 1 Donald and Barbara Zucker School of Medicine at Hofstra/Northwell, 500 Hofstra Blvd, Hempstead, NY 11549 USA; 2 Donald and Barbara Zucker School of Medicine at Hofstra/Northwell and Division of General Internal Medicine, Northwell Health, Hempstead, NY USA; 3 Occupational Medicine, Epidemiology, and Prevention at the Donald and Barbara Zucker School of Medicine at Hofstra/Northwell, Hempstead, USA

## Abstract

**Background:**

There is an increasing shortage of primary care physicians in the U.S. The difficult task of addressing patients’ sociocultural needs is one reason residents do not pursue primary care. However, associations between residents’ perceived barriers to cross-cultural care provision and career interest in primary care have not been investigated.

**Objective:**

We examined residents’ career interest in primary care and associations with resident characteristics and their perceived barriers in providing cross-cultural care.

**Methods:**

We conducted a cross-sectional analysis of a resident survey from the 2018–2019 academic year. We first described residents’ sociodemographic characteristics based on their career interest in primary care (Chi-square test). Our primary outcome was high career interest in primary care. We further examined associations between residents’ characteristics and perceived barriers to cross-cultural care.

**Results:**

The study included 155 family medicine, pediatrics, and internal medicine residents (response rate 68.2%), with 17 expressing high career interest in primary care. There were significant differences in high career interest by race/ethnicity, as Non-White race was associated with high career interest in primary care (*p* < 0.01). Resident characteristics associated with identifying multiple barriers to cross-cultural care included disadvantaged background, multilingualism, and foreign-born parents (all *p*-values< 0.05). There were no significant associations between high career interest in primary care and barriers to cross-cultural care.

**Conclusion:**

Residents from diverse racial/ethnic and socioeconomic backgrounds demonstrated higher career interest in primary care and perceived more barriers to cross-cultural care, underscoring the importance of increasing physician workforce diversity to address the primary care shortage and to improve cross-cultural care.

**Supplementary Information:**

The online version contains supplementary material available at 10.1186/s12909-021-02669-w.

## Introduction

The primary care physician shortage in the United States (U.S.) is increasing with projections estimating a lack of 20,000–50,000 in the next 10–15 years [1, 2]. This has widespread implications, as a robust primary care workforce is associated with improvements in mortality, healthcare access, and quality of care in addition to decreased reliance on safety net institutions [3–7]. Residents cite an inability to address patients’ sociocultural needs during primary care clinical experiences as one reason for not pursuing primary care [8–10]. Cross-cultural care aims to understand the ways in which patients’ backgrounds shape their views of health and can reduce health disparities [11, 12]. An example of cross-cultural care is using an interpreter for a patient with limited English proficiency to improve communication and the patient’s understanding of his or her medical conditions. Residents describe cross-cultural care as important, yet identify barriers, such as language discordance, and lack of preparedness in providing it [13–15]. This suggests barriers to cross-cultural care provision are aspects of primary care that may dissuade trainees from entering the field [16–18].

Exposure to culturally diverse patient populations has been shown to improve trainees’ cultural competency [19–21]. The Accreditation Council for Graduate Medical Education recommends cultural competency training in residency but does not provide specific guidelines, leading to inter-program variance [22, 23]. A sense of social responsibility, such as addressing social determinants of health, has been associated with choosing primary care specialties [24, 25]. Few studies have examined associations between residents’ interest in primary care and perceived barriers to cross-cultural care.

In our study, we identified associations between residents’ sociodemographic and background characteristics and their interest in pursuing primary care. We investigated associations between residents’ characteristics and perceived barriers to cross-cultural care [26, 27]. Finally, we evaluated associations between career interest in primary care and perceived barriers to cross-cultural care provision.

## Methods

### Survey design and study participants

This cross-sectional survey (included in Supplemental Materials) of residents from internal medicine, pediatrics, and family medicine programs at Northwell Health, a large health system consisting of 23 hospitals and groups of physicians providing comprehensive care together [28], was conducted at the beginning of the 2018–2019 academic year. The survey was based on the Cross-Cultural Care Survey [14], which surveyed residents about their preparedness to provide cross-cultural care, training and evaluation in cross-cultural care, and perceived barriers to the provision of cross-cultural care, and similarly covers a variety of topics, including residents’ understanding of and ability to provide cross-cultural care [14]. For this study, we evaluated survey responses pertaining to our primary outcome and main covariates.

### Primary outcome

Our primary outcome was resident career interest in primary care. We identified residents to have high career interest if they indicated 80% or more on a continuous scale, with this cutoff chosen to account for the likelihood that the residents from these specialties may be more inclined to pursue primary care at baseline.

### Covariates

We evaluated residents’ perceived barriers to cross-cultural care as the primary covariate. We identified a barrier when residents answered “moderate problem” or “big problem” versus “small problem” or “no problem”. Additionally, we captured residents’ background characteristics, including disadvantaged background, multilingualism, foreign-born parents, or international medical graduate status.

### Statistical analysis

We first described residents’ sociodemographic characteristics based on high career interest in primary care, performing Chi-square test to determine differences. We then examined differences in residents’ perceived barriers to cross-cultural care based on sociodemographic characteristics and backgrounds. Finally, we evaluated associations between high career interest in primary care and perceived barriers to cross-cultural care. All statistical analyses were conducted using SAS 9.4 (SAS Institute Inc., Cary, NC, USA).

## Results

The study population was 155 residents, as 163/239 eligible residents (response rate 68.2%) from internal medicine, pediatrics, and family medicine completed the survey, with 8 excluded for missing responses to main covariates. The study had a diverse population: 86 female (56%), 57 non-Hispanic White (36.8%), 10 non-Hispanic Black (6.5%), 12 Hispanic (7.7%), 58 non-Hispanic Asian (37.4%), and 18 Other (11.6%). A quarter (27.7%) of residents were from disadvantaged backgrounds. Two-thirds of residents had parents born outside of the U.S. (65.8%), and more than half identified as multilingual (58.7%).

There were 17 residents (11.0%) with high interest in pursuing primary care careers (Table 1). Belonging to a racial/ethnic minority group was associated with high career interest in primary care (*p* < 0.01). Nearly half (44.4%) of family medicine residents had high career interest in primary care versus 2.4% in internal medicine and 17.7% in pediatrics (*p*-value< 0.001).

**Table 1:** Resident sociodemographic characteristics by high career interest in primary care

	All (*n* = 155)	High interest in primary care (*n* = 17)	*P*-value*
Race/Ethnicity			
White, non-Hispanic	57 (36.8%)	5 (29.4%)	0.01
Black, non-Hispanic	10 (6.5%)	4 (23.5%)	
Hispanic	12 (7.7%)	3 (17.6%)	
Asian, non-Hispanic	58 (37.4%)	5 (29.4%)	
Other	18 (11.6%)	0 (0%)	
Male	69 (44.8%)	3 (1.4%)	0.02
Specialty			
Family Medicine	9 (5.8%)	4 (44.4%)	< 0.001
Internal Medicine	84 (54.2%)	2 (2.4%)	
Pediatrics	62 (40.0%)	11 (17.7%)	
Postgraduate year (PGY)			
PGY1	60 (38.7%)	8 (13.3%)	0.65
PGY2	44 (28.4%)	5 (11.4%)	
PGY3+	51 (32.9%)	4 (7.8%)	
Cultural Background			
Disadvantaged background	43 (27.7%)	7 (16.3%)	0.19
Multilingual	91 (58.7%)	11 (12.0%)	0.59
Parents born outside U.S.	102 (65.8%)	11 (10.8%)	0.92
International medical graduate	10 (6.5%)	2 (20.0%)	0.91

*P-values for race/ethnicity, gender, specialty, PGY correspond to comparisons among the high interest group. *P*-values for cultural background correspond to comparisons between those who have high interest and those who do not

We examined associations between residents’ perceived barriers to cross-cultural care and their sociodemographic and background characteristics (Table 2). Postgraduate training year (PGY) was associated with identifying lack of practical experience caring for diverse patient populations, as half (50%) of PGY1 residents selected this compared to only 18.2% of PGY2 and 25.5% of PGY3 residents (*p*-value< 0.05). Residents from disadvantaged backgrounds were more likely to identify inadequate cross-cultural training (*n* = 24, 55.8%) absence of good role models or mentors (*n* = 24, 55.8%), and dismissive attitudes about cross-cultural care among attending physicians (*n* = 20, 46.5%) as barriers (*p*-values< 0.05). Multilingual residents additionally identified dismissive attitudes among colleagues (*n* = 36, 39.6%) as barriers (*p*-value< 0.05). Finally, we examined associations between high interest in primary care careers and perceived barriers to cross-cultural care, which yielded no significant findings.

**Table 2:** Sociodemographic characteristics of residents identifying barriers to cross-cultural care, n (%)

	Identified barriers to cross-cultural care in clinical practice							
	Lack of experience	Lack of time	Inadequate training	Poor access to interpreters	Lack of non-English materials	Lack of mentors	Dismissive attending attitudes	Dismissive resident attitudes
All (*n* = 155)	51 (32.9)	116 (74.8)	65 (41.9)	68 (43.9)	107 (69.0)	62 (40.0)	49 (31.6)	51 (32.9)
High Interest in Primary Care (*n* = 17)	6 (35.3)	14 (82.4)	6 (35.3)	6 (35.3)	12 (70.6)	8 (47.1)	6 (35.3)	6 (35.3)
Race/Ethnicity								
White, non-Hispanic (*n* = 57)	15 (26.3)	45 (78.9)	20 (35.1)	20 (35.1)	39 (68.4)	18 (31.6)	14 (24.6)	15 (26.3)
Black, non-Hispanic (*n* = 10)	4 (40.0)	7 (70)	4 (40.0)	6 (60.0)	7 (70.0)	7 (70.0)	6 (60.0)	6 (60.0)
Hispanic (*n* = 12)	6 (50.0)	7 (58.3)	5 (41.7)	5 (41.7)	10 (83.3)	5 (41.7)	6 (50.0)	6 (50.0)
Asian, non-Hispanic (*n* = 58)	19 (32.8)	44 (75.9)	29 (50.0)	28 (48.3)	37 (63.8)	25 (43.1)	18 (31.0)	18 (31.0)
Other (*n* = 18)	7 (38.9)	13 (72.2)	7 (38.9)	9 (50.0)	14 (77.8)	7 (38.9)	5 (27.8)	6 (33.3)
Male (*n* = 69)	19 (27.5)	66 (77.6)	29 (42.0)	37 (43.5)	43 (62.3)	23 (33.3)	28 (32.9)	24 (34.8)
Specialty								
Family Medicine (*n* = 9)	3 (33.3)	7 (77.8)	4 (44.4)	4 (44.4)	5 (55.6)	4 (44.4)	5 (55.6)	5 (55.6)
Internal Medicine (*n* = 84)	27 (32.1)	61 (72.6)	40 (47.6)	40 (47.6)	56 (66.7)	35 (41.7)	26 (31.0)	29 (34.5)
Pediatrics (*n* = 62)	21 (33.9)	48 (77.4)	21 (33.9)	24 (38.7)	46 (74.2)	23 (37.1)	18 (29.0)	17 (27.4)
Postgraduate year (PGY)								
PGY1 (*n* = 60)	30 (50.0)*	48 (80)	31 (51.7)	31 (51.7)	42 (70.0)	25 (41.7)	23 (38.3)	24 (40.0)
PGY2 (*n* = 44)	8 (18.2)*	30 (68.2)	15 (34.1)	15 (34.1)	30 (68.2)	18 (40.9)	10 (22.7)	9 (20.5)
PGY3+ (*n* = 51)	13 (25.5)*	38 (74.5)	19 (37.3)	22 (43.1)	35 (68.6)	19 (37.3)	16 (31.4)	18 (35.3)
Cultural Background								
From a disadvantaged background								
Yes (*n* = 43)	18 (41.9)	32 (74.4)	24 (55.8)*	24 (55.8)	29 (67.4)	24 (55.8)*	20 (46.5)*	19 (44.2)
No (*n* = 112)	33 (29.5)	84 (75.0)	41 (36.6)*	44 (39.3)	78 (69.6)	38 (33.9)*	29 (25.9)*	32 (28.6)
Multilingual								
Yes (*n* = 91)	33 (36.3)	67 (73.6)	46 (50.5)*	44 (48.4)	66 (72.5)	43 (47.3)*	37 (40.7)*	36 (39.6)*
No (*n* = 64)	18 (28.1)	49 (76.6)	19 (29.7)*	24 (37.5)	41 (64.1)	19 (29.7)*	12 (18.8)*	15 (23.4)*
Parents born outside of the U.S.								
Yes (*n* = 102)	38 (37.3)	76 (74.5)	47 (46.1)	49 (48.0)	71 (69.6)	46 (45.1)*	38 (37.3)*	40 (39.2)*
No (*n* = 53)	13 (24.5)	40 (75.5)	18 (34.0)	19 (35.8)	36 (67.9)	16 (30.2)*	11 (20.8)*	11 (20.8)*
International medical graduate								
Yes (*n* = 10)	2 (20.0)	5 (50.0)*	3 (30.0)	3 (30.0)	5 (50.0)	3 (30.0)	3 (30.0)	3 (30.0)
No (*n* = 145)	49 (33.8)	111 (76.6)*	62 (42.3)	65 (44.8)	102 (70.3)	59 (40.7)	46 (31.7)	48 (33.1)

*All *P*-values < 0.05 and obtained using chi-square test of difference

## Discussion

Prior work has shown the positive effects of patient-physician racial/ethnic and language concordance on patient satisfaction and health outcomes [29–31]. Belonging to a racial/ethnic minority group was associated with interest in primary care, and diverse cultural and linguistic backgrounds were associated with identification of barriers to cross-cultural care. These findings underscore the importance of increasing physician workforce diversity to address the primary care shortage and more competently treat diverse patient populations [32].

Many residents identified barriers to cross-cultural care related to residency training, consistent with prior studies examining cultural competency programming [33, 34]. More PGY1s identified lack of experience providing cross-cultural care as a barrier compared to more senior residents. This indicates that residency training, such as caring for diverse patient populations, curricular initiatives, and working with a diverse group of colleagues, likely influenced this association. These findings warrant further investigation in order to identify which aspects of resident education informed these results.

This study suggests that there is an opportunity to address residents’ perceived barriers to cross-cultural care. One potential approach to increasing residents’ ability to overcome perceived barriers to cross-cultural care is the development of an Entrustable Professional Activity (EPA), or task that is essential for clinical practice [35, 36], that focuses on cultural competency. Cultural competency training exists, yet varies between residency programs and specialties [37–39]. Developing an EPA for cultural competency would be a positive step towards increasing uptake of cultural competency training across residency programs.

Our study had several limitations. The study sample was predominantly non-White residents, which limits generalizability along with our single-center design [40]. The sample includes trainees from specialties likely to produce primary care physicians. Our survey did not capture other possible contributing factors to lower career interest in primary care, such as financial incentives and physician burnout [41]. The cross-sectional study design limits our ability to identify causal relationships.

Residents’ decisions not to pursue primary care careers are multifactorial with one such factor being an inability to adequately address the sociocultural needs of patients [14, 25]. Prior studies demonstrate higher perceived importance of culturally competent care is associated with interest in primary care [42]. Primary care interest was associated with residents’ personal characteristics, such as belonging to a minority group or coming from a disadvantaged background, rather than with residents’ perceived barriers to cross-cultural care. Increasing the racial/ethnic and socioeconomic background diversity may augment the number of residents pursuing primary care careers. With the majority of health outcomes tied to social factors and the growing physician shortage threatening patients’ access to health care, one cannot overemphasize the impact that increasing the number of residents entering primary care can have on improving the health of patients across a spectrum of cultural backgrounds [9, 43].

## Conclusions

Residents from diverse racial/ethnic and socioeconomic backgrounds demonstrated higher career interest in primary care and perceived more barriers to cross-cultural care, underscoring the importance of increasing physician workforce diversity to address the primary care shortage and to improve cross-cultural care.

## Supplementary Information

**Additional file 1.** SURVEY: Cross-Cultural Aspects of Care.

## Data Availability

The data sets used and/or analyzed during the current study are available from the corresponding author upon reasonable request.

## Abbreviations

U.S.: United States
PGY: Postgraduate year
EPA: Entrustable Professional Activity
