# Supplementary material for: Examination of resident characteristics associated with interest in primary care and identification of barriers to cross-cultural care
Source: BMC Med Educ. 2021 Apr 19;21:218. doi: 10.1186/s12909-021-02669-w (PMC8056670; doi:10.1186/s12909-021-02669-w)
Supplement: Supplementary file 1 — Additional file 1. SURVEY: Cross-Cultural Aspects of Care. [file 12909_2021_2669_MOESM1_ESM.doc]

**SURVEY: Cross-Cultural Aspects of Care**

*For the purposes of this questionnaire, we define culturally diverse patients as patients who are members of a culture different from your own.*

|  | No Problem | Small Problem | Moderate Problem | Big Problem |
| --- | --- | --- | --- | --- |
|  | 1 | 2 | 3 | 4 |
| 1. How much of a problem is each of the following when you are delivering cross-cultural care? |  |  |  |  |
| a. Lack of practical experience in caring for diverse patient populations |  |  |  |  |
| b. Lack of time to adequately address cultural issues |  |  |  |  |
| c. Inadequate cross-cultural training |  |  |  |  |
| d. Poor access to medical interpreters when they are needed |  |  |  |  |
| e. Poor access to written materials in other languages, including health education pamphlets, consent forms, etc. |  |  |  |  |
| f. Absence of good role models or mentors for cross-cultural care among the faculty |  |  |  |  |
| g. Dismissive attitudes about cross-cultural care among attending physicians |  |  |  |  |
| h. Dismissive attitudes about cross-cultural care among your fellow colleagues |  |  |  |  |

| 2. Please rate your degree of interest in pursuing a | Not at all | Extremely |
| --- | --- | --- |
| career in primary care | interested On the fence | interested |
| *(Place a mark on the scale above)* | | |
